# Lactate dehydrogenase/albumin ratio as a prognostic factor in severe acute respiratory distress syndrome cases associated with COVID-19

**DOI:** 10.1097/MD.0000000000030759

**Published:** 2022-09-23

**Authors:** Hilal Sipahioglu, Sevda Onuk

**Affiliations:** a Department of intensive Care, Kayseri Training and Research Hospital, Kayseri, Turkey.

**Keywords:** ARDS, COVID-19, lactate dehydrogenase/albumin, mortality

## Abstract

Patients with severe acute respiratory distress syndrome (ARDS) have high mortality rates; therefore, new biomarkers are necessary to predict the prognosis in the early stages. Serum lactate dehydrogenase (LDH) level is a specific marker of lung damage, but it is not sensitive because it is affected by several factors. This study aimed to determine whether the LDH/albumin ratio could be used as a prognostic biomarker in patients with severe ARDS due to COVID 19.

**Methods:**

Tertiary intensive care unit (ICU) patients with severe ARDS and confirmed COVID-19 diagnosis between August 1, 2020, and October 31, 2021, were included. The demographic and clinical characteristics of the patients were recorded from the hospital databases, together with laboratory results on the day of admission to the ICU and the length of stay in the ICU and hospital. LDH/albumin, lactate/albumin, C-reactive protein (CRP)/albumin, and BUN/albumin ratios were calculated. Logistic regression analysis was performed to determine independent risk factors affecting mortality.

**Results:**

Nine hundred and five patients hospitalized in a tertiary ICU were evaluated. Three hundred fifty-one patients with severe ARDS were included in this study. The mortality rate of the included patients was 61.8% (of 217/351). LDH/albumin, lactate/albumin, and BUN/albumin ratios were higher in the nonsurvivor group (*P* < .001). The area under the curve (AUC) from the receiver operating characteristic analysis that predicted in-hospital mortality was 0.627 (95% confidence intervals (CI): 0.574–0.678, *P* < .001) for the LDH/albumin ratio, 0.605 (95% CI: 0.551–0.656, *P* < .001) for lactate/albumin, and 0.638 (95% CI: 0.585–0.688, *P* < .001) for BUN/albumin. However, LDH/albumin ratio was independently associated with mortality in multivariate logistic regression analysis.

**Conclusion:**

LDH/albumin ratio can be used as an independent prognostic factor for mortality in patients with severe ARDS caused by COVID-19.

## 1. Introduction

A novel coronavirus, severe acute respiratory syndrome coronavirus 2, a novel coronavirus, has been identified as the cause of a global respiratory disease outbreak called COVID-19. COVID-19 can be complicated by acute respiratory distress syndrome (ARDS), acute kidney and heart damage, sepsis, septic shock, and multi-organ failure in severe cases. COVID-19 may cause moderate to severe acute respiratory distress syndrome and requires invasive mechanical ventilation for several weeks.^[1,2]^ As patients with severe ARDS have a higher mortality rate, there is a need for new biomarkers for predicting prognosis in the early stages. Endothelial cell damage plays a crucial role in the pathogenesis of ARDS. The serum lactate dehydrogenase (LDH) level is a specific marker of endothelial damage in the lung; however, it is not sensitive because it is affected by several factors.^[3]^

LDH plays an essential role in the body’s tissues in glucose metabolism, catalyzing pyruvate to lactate. Cells release it after damage to their cytoplasmic membranes.^[4]^

Prior research has highlighted the importance of LDH for its ability to detect lung diseases. LDH is a metabolic and prognostic biomarker of immune surveillance, and high levels predict poor outcomes in immunocompromised patients.^[5]^

It increases lactate production, leading to an increase in immunosuppressive cells such as macrophages and dendritic cells. It also inhibits cytolytic cells, including natural killer cells and cytotoxic T-lymphocytes.^[4]^ LDH is usually promoted by the activation and proliferation of T cells.^[6,7]^

A retrospective analysis of a cytotoxic T-lymphocytes antigen-4 antibody that can increase T cell activity and proliferation demonstrated that increased LDH levels indicate a poor outcome^[8]^ According to the autopsy results of patients who died due to COVID-19, T cells play a vital role in this disease.^[9]^ Serum albumin has been used as a prognostic factor for infections because it potentially decreases with infection exacerbation; it is low in patients with sepsis and has been reported to be a crucial factor affecting mortality and prognosis.^[10–12]^

Hypoalbuminemia is also associated with chronic comorbid diseases or lifestyle factors such as smoking, alcoholism, and obesity. Therefore, there have been studies regarding albumin as a single prognostic indicator in infections and as a combination indicator of albumin-based ratios.

Various indices, such as BUN/albumin, lactate/albumin, and C-reactive protein (CRP)/albumin ratios, have been used to predict the severity and prognosis of patients with pneumonia and sepsis.^[13–18]^

Several studies have used the LDH/albumin ratio as a prognostic factor in pneumonia patients.^[19]^ Patients hospitalized with COVID-19 need regular monitoring of vital signs and, where possible, early recognition of the deteriorating patient and biomarkers that predict prognosis. The present study was designed to confirm whether LDH/albumin ratio can be used as an independent prognostic factor in patients with severe ARDS due to COVID-19.

## 2. Material and Methods

Patients hospitalized in the tertiary intensive care unit (ICU) due to COVID-19 between August 1, 2020, and October 31, 2021, were evaluated. Patients were excluded if they had a negative result for polymerase chain reaction, had no albumin level at the time of hospitalization, or had mild or moderate ARDS.

Data were extracted from the electronic medical records of the hospitals. Before collecting the data, the principal researcher established a standardized protocol for data extraction.

This study was approved by the local ethics committee (no.359/2022). The Berlin criteria were used to diagnose ARDS 1. The variables that may be associated with in-hospital mortality were evaluated in this retrospective cohort study. This study included 351 patients with confirmed COVID-19 pneumonia and severe ARDS using the Berlin criteria.

Data were extracted from the electronic medical records of the hospitals. Before collecting the data, the principal researcher established a standardized protocol for data extraction.

This study was approved by the local ethics committee (no. 359/2022). The Berlin criteria were used to diagnose ARDS in the study.^[20]^ The variables that may be associated with in-hospital mortality were evaluated in this retrospective cohort study. This study included 351 patients with confirmed COVID-19 pneumonia and severe ARDS using the Berlin criteria.

Demographic and clinical characteristics of the patients were recorded. The hospital databases were used for hematological and biochemical laboratory results on the day of admission to the ICU, and the length of stay in the hospital ICU was determined in the same manner.

The LDH/albumin, lactate/albumin, CRP/albumin, and BUN/albumin ratios on the first admission day to the ICU were calculated. Kidney Disease: Improving Global Outcomes criteria were used for acute kidney injury (AKI) diagnosis.^[21]^ The acute physiology and chronic health evaluation II (APACHE II) and Sequential Organ Failure Assessment scores were calculated at the end of hospitalization. The primary outcome was in-hospital mortality rate.

## 3. Statistics

Statistical analyses were performed using the IBM SPSS Statistics for Windows (version 22.0; IBM Corp., Armonk, NY). Continuous variables with a normal distribution are presented as mean ± SD deviation and those with a skewed distribution as median (min-max) values. Categorical variables are expressed as numbers (n) and percentages (%). Patients were categorized into two groups: survivors and nonsurvivors. Student *t* test or Mann–Whitney *U* test was used to analyze statistical significance according to the data distribution. Between-group comparisons of categorical variables were performed using the chi-squared test or Fisher exact test.

Forward stepwise binary logistic regression with variables having a *P* value of less than 0.1 in univariate analysis was performed to determine independent factors predicting mortality. Results are presented as odds ratios (OR) and confidence intervals (CI). The linearity was tested by interacting with the logarithmic transformation of each parameter. A receiver operating characteristic (ROC) curve was constructed to determine the best cutoff values of the CRP/albumin, lactate/albumin, LDH/albumin, and BUN/albumin ratios for predicting all-cause mortality.

## 4. Results

Nine hundred and five patients were evaluated in a tertiary ICU. Polymerase chain reaction test results were negative in 119 patients, and 49 patients did not have albumin levels at the time of hospitalization. There were no diagnoses of severe ARDS at the time of admission in the 386 patients. Thus, 351 patients with confirmed COVID-19 pneumonia and severe ARDS were included in the study. The mortality rate of the included patients was 61.8% (of 217/351). The mean age of the included patients was 64.13 ± 12.6 years, and 204 (58.11%) were male.

Patients who died were older than those who survived (*P* < .001). There was no sex difference between the living and dead patients (*P* = .173). The patient demographics and clinical characteristics are shown in Table 1. Patients who died had higher sequential organ failure assessment and APACHE II scores than those who died (*P* < .001). Laboratory information on living and dead patients is summarized in Table 2.

**Table 1:** Demographic and clinical characteristics of severe acute respiratory distress syndrome cases associated with severe COVID-19 patients.

	Overall (n = 351)	Survivors (n = 134)	Nonsurvivors (n = 217)	*P* value
Mean age (yr ± SD)	64.13 ± 12.6	60.56 ± 13.58	66.33 ± 11.45	<.001*
Gender n (%)				
Female	147 (41.88)	50 (37.31)	97 (44.7)	.173
Male	204 (58.11)	84 (62.69)	120 (55.3)	
Comorbidities n (%)				
CKD	28 (7.97)	4 (2.99)	14 (6.45)	.153
Diabetes mellitus	100	45 (33.58)	55 (25.35)	.097
COPD	28 (7.97)	12 (8.96)	16 (7.37)	.595
Hypertension	161 (45.86)	58 (43.28)	103 (47.47)	.445
CAD	72 (20.51)	26 (19.4)	46 (21.2)	.686
Vital Signs				
SBP mm Hg	119.27 ± 24.62	122.41 ± 21.89	118.57 ± 26.19	.277
DBP mm Hg	70.66 ± 17.81	72.64 ± 17.04	69.44 ± 18.2	**.041**
Heart rate/min	112.1 ± 20.52	109.72 ± 21.39	113.58 ± 19.86	**.020**
Respiration rate/min	33.38 ± 6.66	31.91 ± 6.32	34.29 ± 6.72	.001*
Body temperature	36.67 ± 0.92	36.85 ± 1	36.78 ± 0.97	**.049**
PO_2_/FiO_2_	58.55 ± 21.14	61.06 ± 21.66	57 ± 20.72	.058
Glasgow coma scale	13.71 ± 3.01	14.24 ± 2.1513	13.38 ± 3.4	**.021**
SOFA	3.81 ± 1.59	3.31 ± 1.08	4.12 ± 1.77	**<.001**
APACHE II	16.88 ± 5.64	14.59 ± 4.91	18.29 ± 5.62	**<.001**
Mechanical ventilation	277 (78.91)	66 (49.25)	211 (97.24)	**<.001**
AKI, n (%)				
Stage 1	45 (12.82)	13(9.7)	32 (14.75)	**<.001**
Stage 2	36 (10.25)	5 (3.73)	31 (14.29)	
Stage 3	79 (22.50)	4 (2.99)	77 (35.48)	
RRT	78 (22.22)	5 (3.73)	73 (33.64)	**<.001**
Use of vasoactive agent	180 (51.28)	12 (8.96)	168 (77.42)	**<.001**
Median days in ICU (min-max)	13 (2-62)	11.5 (3–55)	14 (2–62)	**.003**
Median days in hospital (min-max)	17 (2-70)	21.5 (4–70)	15 (2–62)	**<.001**

Bold values indicate *P* < .05, statistically significant.

AKI = acute kidney injury, APACHE II = acute physiology and chronic health evaluation II, CKD = chronic kidney disease, COPD = chronic obstructive pulmonary disease, DBP = diastolic blood pressure, ICU = intensive care unit, RRT = renal replacement therapy, SBP = systolic blood pressure, SD = standard deviation, SOFA = sequential organ failure assessment.

**Table 2:** Baseline laboratory parameters of patients included.

	Overall (n = 351)	Survivors (n = 134)	Nonsurvivors (n = 217)	*P* value
Blood urea nitrogen mg/dL	32.28 ± 22.39	26.82 ± 19.03	35.65 ± 23.65	<.001
Creatinine mg/dL	0.9 (0.15–13)	0.85 (0.2–7.87)	0.9 (0.15–13)	.009
Aspartate aminotransferase IU/L	36 (8–1233)	34.5 (9–1233)	39 (8–1052)	.023
Alanine aminotransferase IU/L	28 (2–741)	25 (2–741)	29.5 (4–454	.293
Lactate dehydrogenase U/L	506.5 (57–3401)	496.72 ± 188.09	601.36 ± 348.33	.002
Albumin g/L	32.82 ± 4.7	33.04 ± 4.71	32.68 ± 4.7	.476
Ferritin µg/L	883.5 (26–16,605)	814 (40–7828)	956.5 (26–16,605)	.113
CRP mg/L	107.5 (6.2–480)	105 (8.2–472)	111 (6.2–480)	.456
Procalcitonin µg/L	0.25 (0.01–74)	0.18 (0.01–74)	0.29 (0.01–63)	.019
White blood cell/mm^3^	12,106.06 ± 6185.84	11,808.94 ± 5040.84	12,289.54 ± 6802.07	.780
Neutrophil/mm^3^	10,559.08 ± 5385.96	10,380.22 ± 4743.98	10,669.52 ± 5754.57	.823
Lymphocyte/mm^3^	700 (80–25,000)	740 (260–8680)	670 (80–25,000)	.113
Hemoglobin g/dL	13.02 ± 2.09	13.15 ± 1.99	12.94 ± 2.16	.366
Hematocrit %	38.84 ± 6.13	38.89 ± 5.84	38.81 ± 6.31	.907
Platelets/mm^3^	236,026.78 ± 96,137.4	257,105.97 ± 95,038.6	223,010.14 ± 94,703.18	.001
CK U/L	96.5 (6–4653)	78 (6–3317)	115 (15–4653)	.018
CK-MB U/L	37 (10–344)	34.5 (10–260)	40.5 (12–344)	.051
hs Troponin T ng/L	18 (2–2264)	13.33 (3–1351)	22 (2–2264)	<.001
INR	1.22 ± 0.71	1.08 ± 0.2	1.3 ± 0.88	<.001
Fibrinogen mg/L	6411.81 ± 1803.15	6466.73 ± 2018.45	6376.79 ± 1655.53	.654
D dimer µg/L	1250 (12.13–9000)	1168.5 (12.13–9000)	1340 (110–9000)	.370
Lactate mmol/L	2.1 (0.5–11.4)	1.85(0.5–10)	2.3 (0.5–11.4)	.001
LDH/albumin	15,77 ± 10.48	13.34 ± 8.64	17.28 ± 11.23	<.001
Lactate/albumin	0.656 (0.01–0.52)	0.57 (0.01-0.30)	0.07 (0.02–0.52)	<.001
CRP/albumin	3.14 (0.00–15.48)	2.92 (0.00–15.23)	3.29 (0.00–15.48)	.259
BUN/albumin	0.79 (0.11–5.63)	0.69 (0.11–4.36)	0.89 (0.15–5.63)	<.001

APACHE II = acute physiology and chronic health evaluation II, CK = creatine kinase, CK-MB = creatine kinase myoglobin, CRP = C-reactive protein, LDH = lactate dehydrogenase.

BUN (*P* < .001), LDH (*P* = .002), CRP (although not significant, *P* = .456), and lactate (*P* = .001) levels were higher in patients who died than in those who survived. Albumin levels were lower in patients who died (*P* = .001). The LDH/albumin ratio (*P* < .001) and BUN/albumin ratio (*P* < .001) were higher in deceased patients, while the lactate/albumin ratio (*P* < .001) was lower.

The CRP/albumin ratio (*P* = .259) was similar between the groups (Table 2).

**Figure 1.**
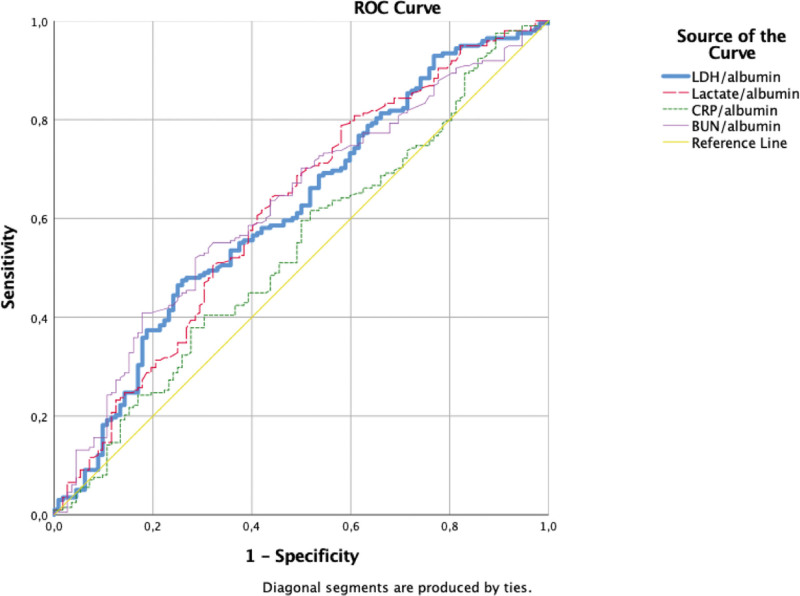
The AUC of the LDH/ALB ratio compared to other albumin-based indexes. AUC = area under the curve, LDH = lactate dehydrogenase.

Figure 1 shows the area under the curve (AUC) for albumin-based ratios used to estimate mortality in patients with severe ARDS.

The AUC for the LDH/albumin ratio was 0.627 (*P* < .001; 95% CI: 0.574–0.678). Other albumin-based ratios of AUCs were 0.638 for BUN/albumin ratio (*P* < .001, 95% CI: 0.585–0.688), 0.605 for the lactate/albumin ratio (*P* < .001, 95% CI: 0.551–0.656), and 0.536 for CRP/albumin ratio (*P* = .256, 95% CI: 0.482–0.589). LDH/albumin sensitivity and specificity were 43% and 78.36%, respectively. A total of 843 was a cutoff value that could predict mortality. The highest sensitivity was observed for lactate/albumin (62.04%), whereas the highest specificity was observed for BUN/albumin ratio (85.07%) (Fig. 1).

In univariate analysis, only LDH/albumin affected hospital mortality among stand-alone and albumin-based ratios. When examined together with other factors affecting mortality by multivariate analysis, the LDH/albumin ratio was found to be an independent risk factor affecting hospital mortality. (*P* < .001, OR = 1.081, 95% CI = 1.035–1.129). Other independent risk factors were advanced age (*P* = .007, OR = 1.046, 95% CI = 1.012–1.081), stage 3 acute renal failure (OR:12.405, 95% CI = 3.177–48.438), high APACHE II (OR = 1.107, 95% CI = 1.032–1.188) and a high number of days in the ICU (OR = 1.458, 95% CI = 1.317–1.614) (Table 3).

**Table 3:** Multiple logistic models for the risk factors of mortality.

	*B*	SE	*P*	OR	95% CI for exp(*B*)	
					Lower	Upper
Constant	−4.545	1.258	<.001			
Age	0.045	0.017	**.007**	1.046	1.012	1.081
DM	−0.971	0.422	**.021**	0.379	0.166	0.866
AKI			**<.001**			
Stage 1	1.774	0.601	**.003**	5.892	1.815	19.133
Stage 2	2.034	0.798	**.011**	7.643	1.599	36.533
Stage 3	2.518	0.695	**<.001**	12.405	3.177	48.437
APACHE II	0.102	0.036	**.005**	1.107	1.032	1.188
LDH/albumin	0.078	0.022	**<.001**	1.081	1.035	1.129
Days in ICU	0.377	0.052	**<.001**	1.458	1.317	1.614
Days in hospital	−0.355	0.050	**<.001**	0.701	0.636	0.774

Bold values indicate *P* < .05, statistically significant.

AKI = acute kidney injury, APACHE II = acute physiology and chronic health evaluation II, DM = diabetes mellitus, ICU = intensive care unit, LDH = lactate dehydrogenase.

## 5. Discussion

The current study showed that the LDH/albumin ratio was an independent risk factor for in-hospital mortality in patients with severe ARDS due to COVID-19. In addition, advanced age, presence of AKI, and ICU stay were independent risk factors affecting hospital mortality. To the best of our knowledge, this is the first study to examine the prognostic effect of LDH/albumin ratio in patients with severe ARDS due to COVID-19.

In the literature, it has been demonstrated that the LDH/albumin ratio independently affects mortality in severe infections requiring intensive care^[19]^ and stroke-related pneumonia^[22]^ in lower respiratory tract infections.^[23]^ High LDH levels are an interesting biomarker associated with poor outcomes in patients with previous viral infections.^[24,25]^

Increased serum LDH levels have been commonly demonstrated in COVID-19 patients and are higher in severely ill patients. Moreover, initial high LDH levels were correlated with the risk of developing ARDS (HR:1.61, 95% CI: 1.44–1.79) and mortality (HR:1.30, 95% CI: 1.11–1.52).^[26]^

All patients in our study had high LDH levels, which were even higher in patients who died (*P* = .002). However, in the logistic regression analysis, LDH alone did not affect the mortality in these patients.

Albumin plays several physiological roles in the body, including the maintenance of osmotic pressure, regulation of vascular permeability, and acid-base balance, and is an anti-inflammatory and antioxidant molecule.^[27,28]^ It has been reported that an albumin level of < 3.5 g/dL affects the 30-day mortality rate in the general patient population hospitalized.^[29,30]^ In a meta-analysis of 90 studies, hypoalbuminemia was associated with prolonged ICU and hospital stays, morbidity, and mortality.^[31]^ In addition, albumin level is lower in patients with severe COVID-19 and has been shown to affect mortality.^[32,33]^

Hoeboer et al showed that LDH is associated with 28-day mortality in ARDS and is valuable in predicting and monitoring the severity and course of acute respiratory distress syndrome.^[34]^

In this study, albumin levels were similar between groups and did not affect mortality.

This result may have been obtained in our study because there was no comparison between severe and mild-to-moderate ARDS or it may be related to the insufficient sample size of the study.

Lee et al reported lower albumin levels and higher LDH and LDH/albumin ratios in patients who did not experience lower respiratory tract infections. In the same study, the LDH/albumin ratio was found to be an independent factor affecting mortality.^[23]^

Mortality is associated with a poor prognosis as lactate levels increase in sepsis patients.^[35]^

In a multicenter study from Italy, although there was no statistically significant relationship with mortality, lactate levels were higher in patients who died in the ICU due to COVID-19 than in those who survived.^[36]^

The lactate/albumin ratio is a good prognostic marker for predicting in-hospital mortality in sepsis patients. Considering patients with septic shock, it has been reported that the lactate/albumin ratio is better than lactate alone in predicting mortality.^[37]^

When the lactate/albumin ratio was examined in sepsis, the present study revealed significantly higher levels in patients who died, but there was no effect on mortality in the multivariate analyses.

Few studies have explored BUN/albumin ratio as a prognostic predictor. These studies reported that BUN/albumin ratio increased as mortality increased in critically ill patients.

When the lactate/albumin ratio based on sepsis was examined, the present study revealed significantly higher levels in patients who died, but this did not affect mortality in multivariate analyses.^[38–40]^

Based on these studies, we examined BUN/albumin ratio in patients with severe ARDS caused by COVID-19. The BUN/albumin ratio was lower in the nonsurvivors than in the survivors (*P* < .001). The specificity of the BUN/albumin ratio was higher than that of other albumin-based ratios. However, it was not an independent risk factor for mortality.

The CRP/albumin ratio has been used as a biomarker for critically ill patients. It was shown to have statistical significance in patients with sepsis compared to patients without sepsis, but it did not affect mortality in sepsis.^[41]^ In our study, neither CRP nor the CRP/albumin ratio affected mortality in patients with severe ARDS.

In this study, advanced age and DM were independent risk factors for mortality in COVID-19 patients, similar to previous studies.^[32,42]^ The incidence of AKI in patients hospitalized for COVID-19 ranged from 46% to 36%. While the incidence of AKI was 76% in patients hospitalized in the ICU, 56% had stage 3.^[43–45]^ In our study, the incidence of AKI was 45.58%, and 22.5% of all the patients had stage 3 AKI. In addition, the presence of AKI is an independent risk factor for mortality.

The APACHE II score is frequently used to measure the severity of disease in the intensive care unit, such that higher scores in our study were also an independent risk factor for mortality.

In conclusion, predictors of mortality are vital in severe ARDS due to COVID-19, which has very high mortality and morbidity. In our study, advanced age, presence of AKI, high APACHE II score, and ICU stay were independent risk factors for mortality. No single parameter affected mortality when laboratory values were examined; however, the LDH/albumin ratio was an independent risk factor for mortality.

## 6. Limitations

Only patients with severe ARDS were included in our study. Better results were obtained if patients with mild to moderate ARDS and those without ARDS were included in the study. In addition, we did not exclude other causes of hypoalbuminemia, and we recorded only the baseline albumin values on the day of hospitalization for all patients.

## Author contributions

**Conceptualization:** Hilal Sipahioglu.

**Data curation:** Hilal Sipahioglu, Sevda Onuk.

**Formal analysis:** Hilal Sipahioglu.

**Funding acquisition:** Hilal Sipahioglu, Sevda Onuk.

**Investigation:** Hilal Sipahioglu.

**Methodology:** Hilal Sipahioglu.

**Project administration:** Hilal Sipahioglu.

**Resources:** Hilal Sipahioglu, Sevda Onuk.

**Software:** Hilal Sipahioglu.

**Supervision:** Hilal Sipahioglu.

**Validation:** Hilal Sipahioglu.

**Visualization:** Hilal Sipahioglu.

**Writing – original draft:** Hilal Sipahioglu, Sevda Onuk.

**Writing – review & editing:** Hilal Sipahioglu.

## References

[1] Chan JF Yuan S Kok KH. A familial cluster of pneumonia associated with the 2019 novel coronavirus indicating person-to-person transmission: a study of a family cluster. Lancet. 2020;395:514–23. 31986261 10.1016/S0140-6736(20)30154-9 PMC7159286

[2] Huang C Wang Y Li X. Clinical features of patients infected with 2019 novel coronavirus in Wuhan, China. Lancet. 2020;395:497–506. 31986264 10.1016/S0140-6736(20)30183-5 PMC7159299

[3] Drent M Cobben NA Henderson RF. Usefulness of lactate dehydrogenase and its isoenzymes as indicators of lung damage or inflammation. Eur Respir J. 1996;9:1736–42. 8866602 10.1183/09031936.96.09081736

[4] Ding J Karp JE Emadi A. Elevated lactate dehydrogenase (LDH) can be a marker of immune suppression in cancer: interplay between hematologic and solid neoplastic clones and their microenvironments. Cancer Biomark. 2017;19:353–63. 28582845 10.3233/CBM-160336 PMC13020749

[5] Kuang ZS Yang YL Wei W. Clinical characteristics and prognosis of community-acquired pneumonia in autoimmune disease-induced immunocompromised host: a retrospective observational study. World J Emerg Med. 2020;11:145–51. 32351646 10.5847/wjem.j.1920-8642.2020.03.003 PMC7183923

[6] Pan L Beverley PC Isaacson PG. Lactate dehydrogenase (LDH) isoenzymes and proliferative activity of lymphoid cells-an immunocytochemical study. Clin Exp Immunol. 1991;86:240–5. 1934592 10.1111/j.1365-2249.1991.tb05803.x PMC1554126

[7] Peng M Yin N Chhangawala S. Aerobic glycolysis promotes T helper 1 cell differentiation through an epigenetic mechanism. Science. 2016;354:481–4. 27708054 10.1126/science.aaf6284 PMC5539971

[8] Dick J Lang N Slynko A. Use of LDH and autoimmune side effects to predict response to ipilimumab treatment. Immunotherapy. 2016;8:1033–44. 27485076 10.2217/imt-2016-0083

[9] Ackermann M Verleden SE Kuehnel M. Pulmonary vascular endothelialitis, thrombosis, and angiogenesis in Covid-19. N Engl J Med. 2020;383:120–8. 32437596 10.1056/NEJMoa2015432 PMC7412750

[10] Yin M Si L Qin W. Predictive value of serum albumin level for the prognosis of severe sepsis without exogenous human albumin administration: a prospective cohort study. J Intensive Care Med. 2018;33:687–94. 28013574 10.1177/0885066616685300

[11] Arnau-Barres I Guerri-Fernandez R Luque S. Serum albumin is a strong predictor of sepsis outcome in elderly patients. Eur J Clin Microbiol Infect Dis. 2019;38:743–6. 30680575 10.1007/s10096-019-03478-2

[12] Godinez-Vidal AR Correa-Montoya A Enriquez-Santos D. Is albumin a predictor of severity and mortality in patients with abdominal sepsis? Cir Cir. 2019;87:485–9. 31448796 10.24875/CIRU.180003903

[13] Hwang YJ Chung SP Park YS. Newly designed delta neutrophil index-to-serum albumin ratio prognosis of early mortality in severe sepsis. Am J Emerg Med. 2015;33:1577–82. 26238097 10.1016/j.ajem.2015.06.012

[14] Wang B Chen G Cao Y Xue J Li J Wu Y. Correlation of lactate/albumin ratio level to organ failure and mortality in severe sepsis and septic shock. J Crit Care. 2015;30:271–5. 25537574 10.1016/j.jcrc.2014.10.030

[15] Luo X Yang X Li J. The procalcitonin/albumin ratio as an early diagnostic predictor in discriminating urosepsis from patients with febrile urinary tract infection. Medicine (Baltim). 2018;97:e11078. 10.1097/MD.0000000000011078 PMC6076169 29995751

[16] Shin J Hwang SY Jo IJ. Prognostic value of the lactate/albumin ratio for predicting 28-day mortality in critically ILL sepsis patients. Shock. 2018;50:545–50. 29461463 10.1097/SHK.0000000000001128

[17] Deng S Gao J Zhao Z. Albumin/procalcitonin ratio is a sensitive early marker of nosocomial blood stream infection in patients with intra-cerebral hemorrhage. Surg Infect (Larchmt). 2019;20:643–9. 31163000 10.1089/sur.2018.260

[18] Gong Y Li D Cheng B. Increased neutrophil percentage-to-albumin ratio is associated with all-cause mortality in patients with severe sepsis or septic shock. Epidemiol Infect. 2020;148:e87. 32238212 10.1017/S0950268820000771 PMC7189348

[19] Jeon SY Ryu S Oh SK. Lactate dehydrogenase to albumin ratio as a prognostic factor for patients with severe infection requiring intensive care. Medicine (Baltim). 2021;100:e27538. 10.1097/MD.0000000000027538 PMC8519202 34731152

[20] Force ADT Ranieri VM Rubenfeld GD. Acute respiratory distress syndrome: the Berlin definition. JAMA. 2012;307:2526–33. 22797452 10.1001/jama.2012.5669

[21] Khwaja A. KDIGO clinical practice guidelines for acute kidney injury. Nephron Clin Pract. 2012;120:c179–84. 22890468 10.1159/000339789

[22] Yan D Huang Q Dai C. Lactic dehydrogenase to albumin ratio is associated with the risk of stroke-associated pneumonia in patients with acute ischemic stroke. Front Nutr. 2021;8:743216. 34604286 10.3389/fnut.2021.743216 PMC8481374

[23] Lee BK Ryu S Oh SK. Lactate dehydrogenase to albumin ratio as a prognostic factor in lower respiratory tract infection patients. Am J Emerg Med. 2022;52:54–8. 34864628 10.1016/j.ajem.2021.11.028

[24] Chen CY Lee CH Liu CY. Clinical features and outcomes of severe acute respiratory syndrome and predictive factors for acute respiratory distress syndrome. J Chin Med Assoc. 2005;68:4–10. 15742856 10.1016/S1726-4901(09)70124-8 PMC7129615

[25] Chiang CH Shih JF Su WJ. Eight-month prospective study of 14 patients with hospital-acquired severe acute respiratory syndrome. Mayo Clin Proc. 2004;79:1372–9. 15544014 10.4065/79.11.1372 PMC7094584

[26] Wu C Chen X Cai Y. Risk factors associated with acute respiratory distress syndrome and death in patients with coronavirus disease 2019 pneumonia in Wuhan, China. JAMA Intern Med. 2020;180:934–43. 32167524 10.1001/jamainternmed.2020.0994 PMC7070509

[27] Hong W Lin S Zippi M. Serum albumin is independently associated with persistent organ failure in acute pancreatitis. Can J Gastroenterol Hepatol. 2017;2017:5297143. 29147647 10.1155/2017/5297143 PMC5632885

[28] Vincent JL Russell JA Jacob M. Albumin administration in the acutely ill: what is new and where next? Crit Care. 2014;18:231. 25042164 10.1186/cc13991 PMC4223404

[29] Lyons O Whelan B Bennett K. Serum albumin as an outcome predictor in hospital emergency medical admissions. Eur J Intern Med. 2010;21:17–20. 20122607 10.1016/j.ejim.2009.10.010

[30] Jellinge ME Henriksen DP Hallas P. Hypoalbuminemia is a strong predictor of 30-day all-cause mortality in acutely admitted medical patients: a prospective, observational, cohort study. PLoS One. 2014;9:e105983. 25148079 10.1371/journal.pone.0105983 PMC4141840

[31] Vincent JL Dubois MJ Navickis RJ. Hypoalbuminemia in acute illness: is there a rationale for intervention? A meta-analysis of cohort studies and controlled trials. Ann Surg. 2003;237:319–34. 12616115 10.1097/01.SLA.0000055547.93484.87 PMC1514323

[32] Li X Xu S Yu M. Risk factors for severity and mortality in adult COVID-19 inpatients in Wuhan. J Allergy Clin Immunol. 2020;146:110–8. 32294485 10.1016/j.jaci.2020.04.006 PMC7152876

[33] Damayanthi H Prabani KIP. Nutritional determinants and COVID-19 outcomes of older patients with COVID-19: a systematic review. Arch Gerontol Geriatr. 2021;95:104411. 33836322 10.1016/j.archger.2021.104411 PMC8010373

[34] Hoeboer SH Oudemans-van Straaten HM Groeneveld AB. Albumin rather than C-reactive protein may be valuable in predicting and monitoring the severity and course of acute respiratory distress syndrome in critically ill patients with or at risk for the syndrome after new onset fever. BMC Pulm Med. 2015;15:22. 25888398 10.1186/s12890-015-0015-1 PMC4381515

[35] Shankar-Hari M Phillips GS Levy ML. Developing a new definition and assessing new clinical criteria for septic shock: for the third international consensus definitions for sepsis and septic shock (Sepsis-3). JAMA. 2016;315:775–87. 26903336 10.1001/jama.2016.0289 PMC4910392

[36] Zanella A Florio G Antonelli M. Time course of risk factors associated with mortality of 1260 critically ill patients with COVID-19 admitted to 24 Italian intensive care units. Intensive Care Med. 2021;47:995–1008. 34373952 10.1007/s00134-021-06495-y PMC8351771

[37] Bou Chebl R Jamali S Sabra M. Lactate/albumin ratio as a predictor of in-hospital mortality in septic patients presenting to the emergency department. Front Med (Lausanne). 2020;7:550182. 33072780 10.3389/fmed.2020.550182 PMC7536276

[38] Ryu S Oh SK Cho SU. Utility of the blood urea nitrogen to serum albumin ratio as a prognostic factor of mortality in aspiration pneumonia patients. Am J Emerg Med. 2021;43:175–9. 32122715 10.1016/j.ajem.2020.02.045

[39] Feng DY Zhou YQ Zou XL. Elevated blood urea nitrogen-to-serum albumin ratio as a factor that negatively affects the mortality of patients with hospital-acquired pneumonia. Can J Infect Dis Med Microbiol. 2019;2019:1547405. 31316681 10.1155/2019/1547405 PMC6604473

[40] Ugajin M Yamaki K Iwamura N. Blood urea nitrogen to serum albumin ratio independently predicts mortality and severity of community-acquired pneumonia. Int J Gen Med. 2012;5:583–9. 22866010 10.2147/IJGM.S33628 PMC3410717

[41] Basile-Filho A Lago AF Menegueti MG. The use of APACHE II, SOFA, SAPS 3, C-reactive protein/albumin ratio, and lactate to predict mortality of surgical critically ill patients: a retrospective cohort study. Medicine (Baltim). 2019;98:e16204. 10.1097/MD.0000000000016204 PMC6617482 31261567

[42] Grasselli G Greco M Zanella A. Risk factors associated with mortality among patients with COVID-19 in intensive care units in Lombardy, Italy. JAMA Intern Med. 2020;180:1345–55. 32667669 10.1001/jamainternmed.2020.3539 PMC7364371

[43] Hirsch JS Ng JH Ross DW. Acute kidney injury in patients hospitalized with COVID-19. Kidney Int. 2020;98:209–18. 32416116 10.1016/j.kint.2020.05.006 PMC7229463

[44] Zhou F Yu T Du R. Clinical course and risk factors for mortality of adult inpatients with COVID-19 in Wuhan, China: a retrospective cohort study. Lancet. 2020;395:1054–62. 32171076 10.1016/S0140-6736(20)30566-3 PMC7270627

[45] Chan L Chaudhary K Saha A. AKI in hospitalized patients with COVID-19. J Am Soc Nephrol. 2021;32:151–60. 32883700 10.1681/ASN.2020050615 PMC7894657

